# Förster resonance energy transfer efficiency measurements on vinculin tension sensors at focal adhesions using a simple and cost-effective setup

**DOI:** 10.1117/1.JBO.28.8.082808

**Published:** 2023-07-11

**Authors:** Camille Dubois, Ludivine Houel-Renault, Marie Erard, Nada N. Boustany, Nathalie Westbrook

**Affiliations:** a Université Paris-Saclay, Institut d’Optique Graduate School, CNRS, Laboratoire Charles Fabry, Palaiseau, France; b Université Paris-Saclay, Institut des Sciences Moléculaires d’Orsay, CNRS, Centre de Photonique pour la Biologie et les Matériaux, Orsay, France; c Université Paris-Saclay, Institut de Chimie Physique, CNRS, Orsay, France; d Rutgers University, Department of Biomedical Engineering, Piscataway, New Jersey, United States

**Keywords:** Förster resonance energy transfer, tension sensor, focal adhesion, vinculin

## Abstract

**Significance:**

Forces inside cells play a fundamental role in tissue growth, affecting important processes such as cancer cell migration or tissue repair after injury. Förster resonance energy transfer (FRET)-based tension sensors are a remarkable tool for studying these forces and should be made easier to use.

**Aim:**

We prove that absolute FRET efficiency can be measured on a simple setup, an order of magnitude more cost-effective than a standard FRET microscopy setup, by applying it to vinculin tension sensors (VinTS) at the focal adhesions of live CHO-K1 cells.

**Approach:**

Our setup located at Université Paris-Saclay acquires donor and acceptor fluorescence in parallel on two low-cost CMOS cameras and uses two LEDs for rapid switching of the excitation wavelength at a reduced cost. The calibration required to extract FRET efficiency was achieved using a single construct (TSMod). FRET efficiencies were measured for VinTS and the tail-less control VinTL, lacking the actin-binding domain of vinculin. Measurements were confirmed on the same cell type using a more standard intensity-based setup located at Rutgers University.

**Results:**

The average FRET efficiency of VinTS (22.0%±4%) over more than 10,000 focal adhesions is significantly lower ($p<10^{-6}$) than that of VinTL (30.4%±5%), our control that is insensitive to force, in agreement with the force exerted on vinculin at focal adhesions. Attachment of the CHO-K1 cells on fibronectin decreases FRET efficiency, thus increasing the force, compared with poly-lysine. FRET efficiency for the VinTL control is consistent with all measurements currently available in the literature, confirming the validity of our measurements and hence of our simpler setup.

**Conclusions:**

Force measurements, resolved spatially inside a cell, can be achieved using FRET-based tension sensors with a cost effective intensity-based setup. This will facilitate combining FRET with techniques for applying controlled forces such as optical tweezers.

## 1 Introduction

**Fig. 1:**
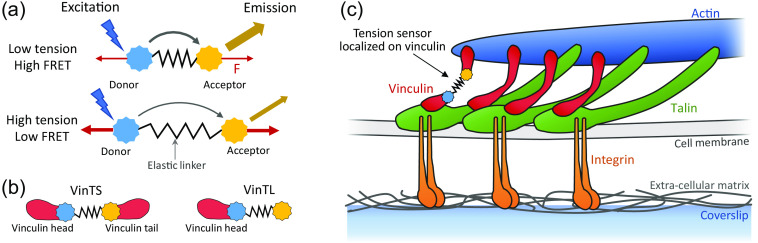
(a) The tension sensor module (TSMod) consists of two fluorophores of the FRET pair mTFP1-mVenus separated by an elastic linker sequence. When force is exerted across the tension sensor, FRET efficiency decreases. (b) The VinTS^7^ construct consists of TSMod inserted between the vinculin’s head and tail. Vinculin tail-less (VinTL) is a construct that lacks the vinculin tail. (c) Simplified diagram of a focal adhesion (FA) showing integrins, talins, vinculins with and without the tension sensor, an actin filament, the cell membrane, and the extracellular matrix. Vinculin is recruited and activated at focal adhesions (FAs) with its head attached to talin and its tail to actin.

Förster resonance energy transfer (FRET) measurements are a unique way to measure nanometer-scale distances between two fluorophores, a donor and an acceptor, without the need for high resolution imaging. When the donor and acceptor pair are connected with an elastic linker subjected to tension, the distance measurement can be translated into a force measurement, with picoNewton sensitivity.^1^^–^^6^ These FRET tension sensors have been inserted inside proteins involved in cell attachment to the extra-cellular matrix, in particular inside vinculin^7^ (Fig. 1). They open the way to imaging variations of these forces with external stimuli, either the rigidity of the substrate on which cells are attached^8^ or applied forces by an optical tweezer,^9^ and can be combined with other methods, such as micropillar-based traction force sensors, to compare molecular tension with traction forces at the cellular level.^10^ Although the variation in FRET efficiency on a given setup is a good way to measure a relative change in force, an absolute measurement of the FRET efficiency is required if an absolute force measurement is needed or if comparison on the same FRET sensor between different setups is desirable. The direct measurement of FRET efficiency is usually achieved with fluorescence lifetime measurements (FLIM); however, such experiments typically require a costly scanning microscopy setup with a short-pulse laser and a detector able to measure nanosecond lifetimes. Wide-field imaging using either frequency-domain or time-domain FLIM circumvents point scanning but requires specialized and costly excitation sources and cameras capable of detecting signals modulated at high frequencies (FD-FLIM) or of resolving photon arrival times (TD-FLIM).^11^^–^^14^ Sensitized FRET measurements, based on measurements of fluorescence intensities, can be done on simpler setups, providing images with a large field of view without scanning. However, they require a calibration procedure to take into account bleedthrough between fluorescence channels. They can then provide a FRET efficiency that can be compared to other experiments and not just a relative FRET index,^15^ specific to a given setup. Here we show that accurate FRET efficiency measurements on tension sensors can be made on a wide-field setup that is significantly less complex and an order of magnitude less costly than most other setups designed for FRET microscopy. Such setups typically rely on commercial microscopy platforms equipped with high-end complementary metal oxide semiconductor (CMOS) or charge coupled device (CCD) cameras and costly filter wheels that are specifically made to fit such commercial platforms. Here, we circumvent the use of these less versatile and more costly components without a loss of measurement sensitivity and provide a modular platform that can be designed and easily modified to allow for additional measurements in conjunction with FRET, such as the use of optical tweezers in the context of mechanobiology studies. We take advantage of the progress in light emitting diode (LED) sources and in CMOS cameras and simultaneously capture the donor and acceptor fluorescence on two standard cameras, avoiding any moving parts in the system. After a calibration procedure following a prior work,^16^ we measured 22.0% average FRET efficiency for the vinculin tension sensor (VinTS)^7^ on focal adhesion sites of CHO-K1 cells, compared with 30.4% for the tail-less control VinTL (Fig. 1). We prove the validity of this simplified setup located in Université Paris-Saclay through the same measurements performed on a more standard sensitized setup located at Rutgers University^17^ and also by comparison with several such measurements reported in the literature for other cell types and other methods, including FLIM.

## 2 Methods

### 2.1 Optical Setup

**Fig. 2:**
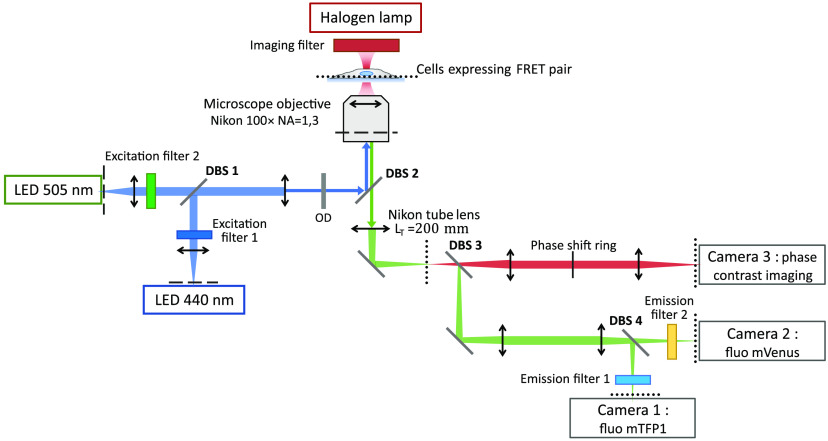
Optical setup: both LEDs are conjugated with the back focal plane of the microscope objective (dashed line). The sample is imaged on three cameras: cameras 1 and 2 for fluorescence of the donor and acceptor, respectively, and camera 3 for phase contrast (dotted line). Illumination for the phase contrast image is done with a halogen lamp with a red filter (high pass >590  nm), an annulus ring in the condenser, and a phase ring deported on the imaging path. All dichroic and filters references are listed in Fig. 3(a).

**Fig. 3:**
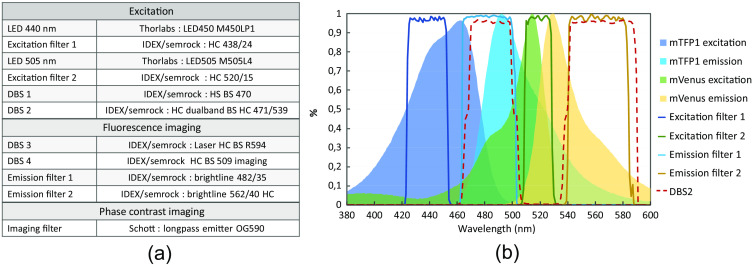
(a) List of the filters. (b) Spectra of mTFP1 and mVenus excitation and emission and transmission of the filters.

Our setup is depicted in Fig. 2. Two non-polarized LEDs excite the sample : a blue LED at 440 nm to excite the donor fluorophore mTFP1 and a green LED at 505 nm to excite the acceptor mVenus (see Fig. 3(a) for reference). Both are collimated using aspheric lenses with 32-mm focal lengths (Thorlabs ref ACL-50832U-A). The current in each LED can be adjusted independently to reach the desired illumination intensity in the sample plane. A neutral density filter is inserted on the common path to reduce both illumination intensities. Intensities between $13.5\;\mathrm{mW}/{\mathrm{mm}}^{2}$ and $41\;\mathrm{mW}/{\mathrm{mm}}^{2}$ for the blue LED (440 nm) and between $19\;\mathrm{mW}/{\mathrm{mm}}^{2}$ and $55.5\;\mathrm{mW}/{\mathrm{mm}}^{2}$ for the green LED (505 nm) were used in the different measurements. Fluorescence is collected through a microscope objective (Nikon Plan Fluor 100×, NA=1.3, oil immersion) and the donor and acceptor emissions are detected simultaneously on two CMOS cameras (Basler acA2040-55  μm, 2048×1536  pixels, 3.45  μm pixels, quantum efficiency 65% at 550 nm), one for the donor and one for the acceptor. The two lenses used to relay the image from the exit port of the microscope to the two cameras are achromats with focal lengths of 150 mm (Thorlabs AC254-150A).

For each FRET measurement, we acquire three images in two steps.

- First, we turn on the blue LED (440 nm) for a time $t_{D}$ to excite the donor and acquire simultaneously two images: DD (Donor excitation and Donor detection) on camera 1 and DA (Donor excitation and Acceptor detection) on camera 2.
- Then we turn on the green LED (505 nm) for a time $t_{A}$ to excite directly the acceptor and acquire the AA (Acceptor excitation and Acceptor detection) image on camera 2.

The spectral transmission bands of the excitation and detection filters are shown in Fig. 3(b), overlayed on the absorption and emission spectra of the two fluorophores. References of all filters and beamsplitters used in the setup are shown in Fig. 3(a). Phase contrast imaging is performed with red light illumination (a halogen lamp with a OG590 Schott filter) to avoid photobleaching of the fluorophores during initial sample focusing. An annulus ring is placed in the back focal plane of the condenser and is conjugated with an external phase ring on the imaging path to avoid modifying the microscope objective. Adjustment of the relative position of the two cameras is accomplished using fluorescent microbeads (Fluoresbrite^®^YG Microspheres 0.50  μm, Polysciences) to overlap the two images with the same magnification. Due to chromatic aberration, this position does not correspond to good focusing on both cameras. A displacement of 200 nm of the sample could recover a sharp focus, which gives an estimate of the overall chromatic aberration of the fluorescence imaging path between the 482 and 562 nm detection bands. To avoid moving the sample, we adjusted the distance between the dichroic beamsplitter DBS4 and camera 1 by 2 mm (200 nm multiplied by the square of the 100× lateral magnification) to reach precise focusing on the two cameras. Additional fine tuning of this distance is done using images of the focal adhesions themselves. The magnification is not exactly the same on both cameras and is corrected during image processing (DD is 2% smaller than DA and AA). Compared with a standard setup with a single camera, we do not need to move the sample between DD and AA acquisitions to compensate for the chromatic aberration.

We characterized the bleedthrough between channels using cells transfected with acceptor only or donor only. The cells were isolated from the background by thresholding, and pixel-by-pixel heatmaps of DA versus AA or DA versus DD were plotted. A linear fit of these heatmaps gave a factor a=0.054 for the direct excitation of the acceptor when illuminating with the blue LED at 440 nm (measured with the acceptor only construct in the cell) and a factor d=0.384 for the long tail of the donor fluorescence above 550 nm that passes through the acceptor detection channel (measured with the donor only construct). These lead to a corrected FRET intensity of

$$F_{c}=\mathrm{DA}-a\;\mathrm{AA}-d\;\mathrm{DD}.$$ (1)

To simplify notations, we call DA, AA, and DD the intensities of the corresponding images.

The FRET efficiency measurements made on this simplified setup (Fig. 2) located at Paris-Saclay are validated against the same measurements performed on a more standard FRET microscopy setup located at Rutgers utilizing filter wheels and a high-end scientific CMOS camera^17^ (PCO Edge 4.2bi, 2048×2048  pixels, 6.5  μm pixels, quantum efficiency 95% at 550 nm, cooled at −25°C).

### 2.2 Sample Preparation

Chinese Hamster ovary cells (CHO-K1) (ATCC CCL-61) were grown and maintained at 37°C, 5% ${\mathrm{CO}}_{2}$ in high glucose Dulbecco’s Modified Eagle’s Medium supplemented with 10% fetal bovine serum, 1% penicillin/streptomycin, and 1% L-glutamine (all from Gibco) for Paris-Saclay experiments or F12K (ATCC) supplemented with 10% fetal bovine serum (Gemini) at Rutgers. Cells were plated at $10,000/{\mathrm{cm}}^{2}$ if transfected the next day or at $5,000/{\mathrm{cm}}^{2}$ if transfected 3 days after plating. Cells were seeded on 25 mm diameter glass coverslips (1.5H Marienfeld Superior), previously cleaned with nitric acid (18-mm coverslips cleaned with chromic sulfuric at Rutgers), rinsed 10 times with sterile water, then incubated 1 min in alcohol, and air dried. Coverslips were then incubated with 10  μg of poly-L-lysine from sciences sell [poly-D-lysine (PL) at Rutgers, P0899, Sigma] for 30 min at 37°C and then washed three times with sterile water or incubated with 10  μg of FN (F0895, Sigma) for 30 min at 37°C. Cell transfections were performed following the Lipofectamine LTX protocol (Invitrogen) with a DNA ratio of 3.75  μg/5  μl of plasmid/lipofectamine per coverslip (plasmid descriptions are given in Table S1 in the Supplementary Material). Cells are imaged between 24 and 48 h after transfection in live cell imaging solution (Invitrogen) or growth medium in Paris-Saclay or Leibovitz’s + 10% serum at Rutgers.

### 2.3 Image Processing

The three raw images (DD, DA, and AA) are first background corrected by subtracting the mean value of a background region selected manually far from any cell. Each background-substracted image is flat-field corrected to account for the illumination profile of each LED that is not perfectly uniform over the full field of view.^18^^,^^19^ A reference image is obtained for each LED by averaging and smoothing 15 to 20 images of a microscope slide with uniform fluorescence (fluorescent marker).

To compensate for translation and rotation between the two cameras and correct for the change in magnification due to chromatic aberrations, DD has to be registered to perfectly match pixel-by-pixel with DA and AA.

The transformation is calculated for each coverslip tested and applied to all of the DD images. DA and AA are already perfectly overlaid because they are acquired on the same camera without any mechanical movement between the acquisitions. The non-background pixels selected from cells expressing mTFP1, mVenus, or TSMod are segmented using a thresholding. For VinTS and VinTL, simple thresholding cannot extract focal adhesions as they appear over a fluorescence background from the cytoplasm. A binary mask to isolate focal adhesions is calculated based on the subtraction of a spatially varying background calculated with a rolling ball algorithm (ball diameter = 20 pixels),^20^ followed by a Gaussian blur (σ=3  pixels) and thresholding. Only the FAs consisting of at least 30 pixels ($\approx 0.04\;\mu m^{2}$ in the object plane) are kept for the analysis. FRET efficiencies are averaged over each segmented FA. Details of the image processing are given in the Supplementary Material.

## 3 Results

### 3.1 Calibration

To obtain an absolute FRET efficiency measurement that can be compared to other experiments, calibration of both the Paris-Saclay and Rutgers sensitized emission setups was done using a single FRET construct TSMod, following the method described in Menaesse 2020.^16^ This TSMod construct is the one that will be inserted in the vinculin protein to create the tension sensor VinTS. Its FRET efficiency has been previously measured to be 28.6%,^21^ but we measured its value in our CHO-K1 cells as explained further in this section. We determined the calibration factors^22^^–^^25^ G and k specific for each of our imaging setups. The given constructs and setups are listed as follows.

• The following relationship is used to determine G:
  $$\frac{F_{c}}{\mathrm{AA}}=G\frac{E}{1-E}\frac{\mathrm{DD}}{\mathrm{AA}}.$$ (2)

**Fig. 4:**
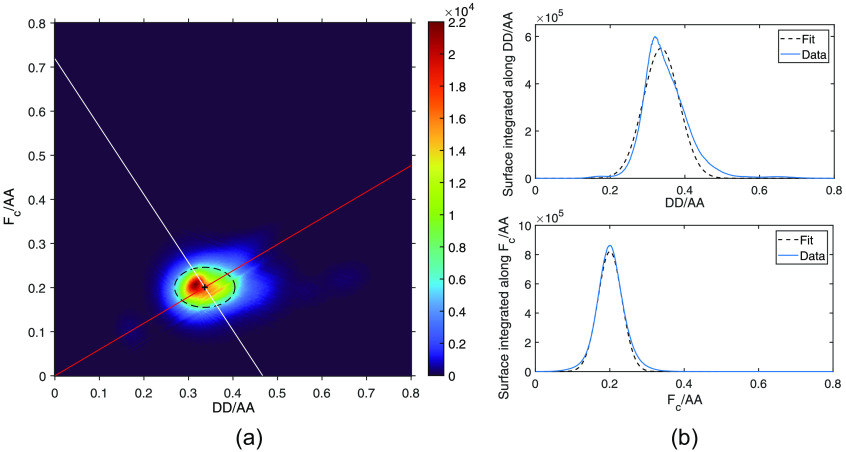
(a) Pixel-by-pixel heatmap of Fc/AA versus DD/AA of CHO-K1 cells expressing TSMod. 269 cells from 6 samples with exposure times of 100 or 200 ms and ND filter of OD 1 (around 48 millions pixels). The pixel values are fitted with a 2D Gaussian: $f(x,y)=A\;\exp(-{\left((x-x_{0})/w_{x}\right)}^{2}-{\left((y-y_{0})/w_{y}\right)}^{2})$. $R^{2}=0,967$. The black cross represents the center of the fitted Gaussian, and the dashed ellipse is the 95% confidence bound of the Gaussian. The center of the Gaussian is given by $x_{0}=0.3366\pm 1.2e^{-4}$ and $y_{0}=0.2005\pm 8.0e^{-5}$. The red and white lines represent Eqs. (2) and (3), respectively. (b) Projections of the 2D histogram along x and y axes.

$F_{c}$ is the FRET corrected from bleedthroughs [see Eq. (1)], DD is the donor emission when donor is excited, and AA is the acceptor emission when the acceptor is excited. From the slope of the $F_{c}/\mathrm{AA}$ versus DD/AA plot, and the FRET efficiency E measured by FLIM, we obtain the G calibration factor [red line in Fig. 4(a)].

• Then, because we use constructs with a fixed donor to acceptor ratio of 1:1, we obtain the calibration factor k from
  $$\frac{F_{c}}{\mathrm{AA}}=-G\frac{\mathrm{DD}}{\mathrm{AA}}+G\;k.$$ (3)

Tracing the line of slope −G passing through the data points, we get k as the intersection with the axis where $\frac{F_{c}}{\mathrm{AA}}=0$ [white line in Fig. 4(a)].

Two phenomena can bias the calibration: photobleaching and a high concentration of fluorophores in the cells. First, to avoid photobleaching during focusing, cell images are focused using phase contrast with red light illumination. We quantified photobleaching with each LED on cells expressing mTFP1 or mVenus only. With the OD = 1 that was used for the calibration, the illumination intensities are $19.0\;\mathrm{mW}/{\mathrm{mm}}^{2}$ for the blue LED (440 nm) and $13.5\;\mathrm{mW}/{\mathrm{mm}}^{2}$ for the green LED (505 nm). The photobleaching decay half-time was 450 s for mTFP1 and 21 s for mVenus (Fig. S1 in the Supplementary Material). Because we used exposure times of 100 or 200 ms, photobleaching is negligible. Second, cells expressing very high concentration of fluorophores have a higher FRET index $F_{c}/\mathrm{AA}$, probably due to intermolecular FRET as a consequence of molecular crowding. To address this issue, we chose a range of intensity over which the cells have constant FRET index over $\mathrm{AA}/t_{A}$ (AA intensity normalized to exposure time) while keeping a good signal to noise ratio (Fig. S2 in the Supplementary Material).

The corrected FRET and donor intensities both normalized by acceptor intensity are plotted pixel-by-pixel for the non-background pixels of 269 TSMod cells [Fig. 4(a)]. Data are fitted with a 2D Gaussian model.

**Fig. 5:**
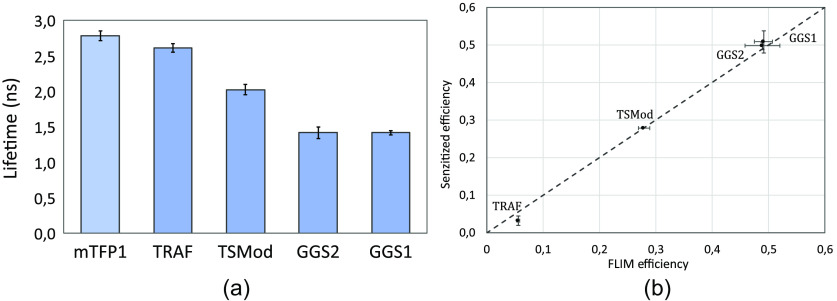
(a) Lifetime measurements for mTFP1 (37 cells), TRAF (20 cells), TSMod (15 cells), GGS2 (26 cells), and GGS1 (8 cells). Average lifetime values ± standard deviation are respectively : 2.78±0.07  ns, 2.62±0.06  ns, 2.02±0.07  ns, 1.42±0.08  ns, and 1.42±0.03  ns. (b) Sensitized FRET efficiencies measured on the Paris-Saclay setup for TRAF (26 cells), GGS2 (8 cells), and GGS1 (28 cells), based on the calibration using TSMod only. The FLIM efficiencies are calculated from the lifetimes shown in (a). Average efficiencies values ± standard deviation. The dashed line has a slope of 1, showing that the sensitized and FLIM efficiencies are the same.

As a reference for FRET efficiencies in our CHO-K1 transfected cells, we used a FLIM setup^26^ to measure the donor lifetime for TSMod. Measured lifetimes $\tau_{D}$ for donor (mTFP1) only and $\left\langle \tau_{\mathrm{DA}}\right\rangle$ (weighted average of two contributions) for donor in the presence of acceptor for TSMod are shown in Fig. 5(a). The FRET efficiency of TSMod deduced from these measurements ($E=1-\frac{\left\langle \tau_{\mathrm{DA}}\right\rangle }{\tau_{D}}$) is 27.9±1.0%, in good agreement with published values for this construct.^21^ From the coordinates of the center of the Gaussian and this TSMod FRET efficiency of 27.9±1.0% from our FLIM measurement, we obtain : G=1.54±0.08 and k=0.47±0.03. The same method was used to calibrate the Rutgers setup, and it gave G=2.97±0.10 and k=2.14±0.08 (see corresponding heatmap in Fig. S3 in the Supplementary Material). The uncertainty on G is mostly due to the uncertainty on the FRET efficiency of TSMod measured by FLIM.

As a confirmation of the calibration of our Paris-Saclay setup, we measured the FRET efficiencies of constructs with low (TRAF) and high (GGS1 and GGS2) FRET values (see Table S1 in the Supplementary Material for all plasmids). Figure 5(b) shows that the efficiencies calculated with our calibration factor G=1.54±0.08 coincide with the efficiencies deduced from lifetime measurements for these constructs performed with our FLIM setup [Fig. 5(a)]. They also agree with published values.^25^ This agreement confirms the validity of our calibration with TSMod only.

### 3.2 Vinculin Tension Sensor

**Fig. 6:**
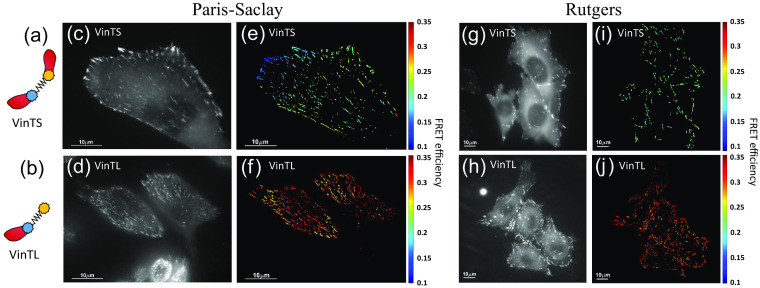
(a) VinTS consists of TSMod inserted between the vinculin head and tail. (b) Vinculin tail-less sensor (VinTL) is a control construct only linked to the vinculin head. (c), (d), (g), and (h) Fluorescence images in the acceptor channel of cells expressing VinTS and VinTL. (e), (f), (i), and (j) The FRET efficiencies averaged over the segmented FAs of cells expressing VinTS and VinTL. Images (c-f) are taken on the Paris-Saclay setup with an exposure time of 1900 ms. Images (g-j) are cropped images of 1100×1100  pixels from the total field of view of 2048×2048  pixels, taken on the Rutgers setup with an exposure time of 1000 ms.

**Fig. 7:**
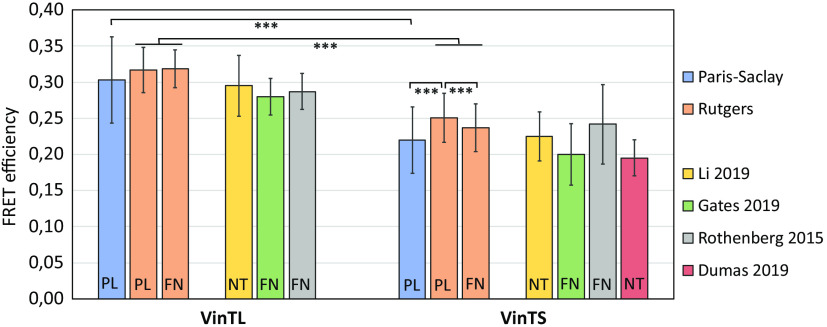
FRET efficiencies measured over FAs of cells expressing VinTS or VinTL. We repeated the same experiment on Paris-Saclay and Rutgers setups calibrated as explained in 3.1. FRET efficiency is averaged over each segmented FA. Paris-Saclay: CHO-K1 cells seeded on poly-L-lysine. Rutgers: CHO-K1 cells seeded on PL or FN. Our results can be compared to other measurements: Li:^28^ lifetime measurements on TMD cells. Gates:^25^ ratiometric measurements on vinculin -/- MEFs cells. Rothenberg:^21^ ratiometric measurements on Vin -/- MEFs cells. Dumas:^29^ lifetime measurements on iBMK cells. The coverslip treatment of each experiment is given by PL: polylysine, FN: fibronectine, or NT: no treatment. Mean ± standard deviation. *** denotes significance of $p<10^{-3}$ by ANOVA followed by multiple comparison test.

FRET efficiency measurements of VinTS and VinTL on the focal adhesion sites of CHO-K1 cells plated on poly-L-lysine, using the simplified Paris-Saclay setup (Fig. 2), are shown in Figs. 6 and 7 (blue bars). Figure 6 shows examples of raw AA images (left) and corresponding segmented images in which FRET efficiency is calculated based on the calibration described above and averaged over each focal adhesion site for VinTS (top panels) and VinTL (bottom panels). FRET efficiency is higher for VinTL, whereas for VinTS the presence of force reduces FRET efficiency. Average FRET efficiencies over 10,000 adhesion sites from 60 cells (VinTL) and 19,000 adhesion sites from 87 cells (VinTS) are shown in the blue bars of Fig. 7. The VinTL FRET efficiency is 30.4%±5%, slightly above the TSMod efficiency of 27.9%. For VinTS on poly-L-lysine, the average efficiency is significantly lower, at 22.0%±4% (p-value $<10^{-3}$ by one-way ANOVA). Uncertainties are standard deviations on all adhesion sites.

Compared with the TSMod, mVenus, and mTFP1 experiments, higher illumination intensities and longer exposure times were required for the VinTS and VinTL experiments due to the lower fluorophore concentration on the focal adhesion sites. An OD = 0.6 was used and yielded $55.5\;\mathrm{mW}/{\mathrm{mm}}^{2}$ for the blue LED (440 nm) and $41.0\;\mathrm{mW}/{\mathrm{mm}}^{2}$ for the green LED (505 nm), and exposure times were increased to 1900 ms. In our analysis, we discarded adhesion sites that had low fluorescence signals (AA lower than 5 gray levels with our exposure time of 1.9 s). This situation corresponds to a signal over background equal to 1 on the DA image, the one with the highest background compared with DD and AA, due most likely to the autofluorescence of the sample. We checked that the choice of this threshold changes the value of the average efficiency by <2% for VinTL (see Fig. S10 in the Supplementary Material).

Assuming a supralinear dependence of the photobleaching rate with the illumination intensity as reported in Ref. 27 (exponent α=1.27 for mVenus with widefield LED illumination at 505 nm), we estimated the photobleaching half-time to be $t_{A,1/2}=5.1\;s$ for the green LED (505 nm) at $41\;\mathrm{mW}/{\mathrm{mm}}^{2}$. Following Ref. 23, we corrected our FRET efficiencies by $E_{\mathrm{corr}}=E\times 2^{\frac{t_{\text{exp}}/2}{t_{A,1/2}}}$ with $t_{A,1/2}$ being the acceptor photobleaching half-time. We divided the exposure time by 1/2 to take into account that the signal integrated over an exposure time while photobleaching is approximately the same as a signal with no photobleaching at half the exposure time. This approximation is valid to within 0.2% in our conditions. For our exposure time of 1.9 s, this correction increases the FRET efficiencies by a factor of 1.12 and has been taken into account in the values for VinTS and VinTL given above.

Using the same protocol, the same cells and same image processing method, we repeated the same measurements on the Rutgers setup (described in Ref. 17). The excitation intensity for the acceptor was much lower ($0.8\;\mathrm{mW}/{\mathrm{mm}}^{2}$) with an exposure time of 1 s, so no correction for photobleaching was necessary. In addition, we tested the cells attachment on either PL or fibronectin (FN) to allow for comparisons with previously published values found in the literature. The FRET efficiency of the tension-insensitive VinTL control (Fig. 6) was in close agreement on both setups: 31.7%±3% on PL (Rutgers) and 30.4%±5% on PL (Paris-Saclay) (p-value= 0.99 by two-way ANOVA). VinTL FRET efficiency was also not significantly affected by the different substrate treatment: 31.7%±3% on PL and 31.9%±3% on FN (p-value = 0.96). However, the different substrate coatings lead to a significant difference in FRET efficiency for VinTS (25.1%±3% on PL and 23.7%±3% on FN, p-value $<10^{-3}$). The orange bars in Fig. 7 show those results for VinTL and VinTS. The FRET efficiency of VinTS was higher on the Rutgers setup (25.1%±3%) compared with the Paris-Saclay (22.0%±4%) setup (p-value $<10^{-3}$). Thus the absolute FRET efficiency difference between VinTL and VinTS was 8.4% on the Paris-Saclay setup versus 6.6% on the Rutgers setup.

## 4 Discussion

In this paper, we have described a simplified and cost-effective setup to facilitate FRET measurements that we built in Paris-Saclay and validated it against measurements made on a more standard FRET microscopy setup located at Rutgers. These measurements were performed at focal adhesion sites of CHO-K1 cells on a tension sensor inserted between the head and tail of vinculin (VinTS) and compared with a force insensitive tail-less control (VinTL). Small changes in FRET efficiency of VinTS can be translated into force variations. Variation of these forces depending on the surface treatment on which the cells are plated was observed. First, we observed that all VinTL efficiencies were similar on the Paris Saclay (blue bar) and Rutgers setups with both surface treatments (orange bars). This confirmed the validity of our calibration procedure to obtain FRET efficiencies, and that VinTL efficiency is not affected by force. To extend the comparison, we added in Fig. 7 values for VinTL extracted from the literature: all of them agreed reasonably well around 30% FRET efficiency, whether they were obtained from lifetime (Li et al.^28^ and Dumas et al.^29^) or sensitized measurements (Rothenberg et al.^21^ and Gates et al.^25^). VinTS FRET efficiencies, both on our two setups and in the literature, are lower than the VinTL, which is expected because VinTS is sensitive to force. However, the VinTS FRET efficiency measurements differ, ranging from 20% to 25%, which may correspond to different forces exerted on the focal adhesion sites depending on the cell type and on the surface treatment of the culture substrate. The fact that VinTS FRET on the Paris-Saclay setup was 1.8% lower than VinTS FRET measured on the Rutgers setup using the same cells on poly-lysine, while the VinTL FRET efficiencies remained in close agreement, suggested that slight changes in culture conditions could affect molecular tension at adhesion sites. Still the difference between VinTL and VinTS FRET was consistent across multiple laboratories and different substrate coating conditions, supporting the validity of these probes as standards for molecular tension measurements and for testing our setup.

Unlike two-state biosensors, which toggle between high and low FRET efficiency with a large difference in FRET (absolute FRET efficiency difference on the order of 30%, for example in Ref. 30), the FRET efficiency of VinTS can vary continuously over the range of vinculin tension experienced in the cell. Thus the difference between VinTS and VinTL FRET efficiency was typically small by comparison (absolute efficiency difference on the order of <10%, as seen in Fig. 7) and can be challenging to measure.

Our results on these tension sensors show that our simple optical setup has proven capable of measuring FRET efficiencies in live cells with adequate sensitivity to resolve small changes in FRET efficiency. Replacing a single mercury-arc or xenon source by two separate LED not only reduces cost and increases lifespan but also makes it easier to adjust independently the two excitations to adjust to the characteristics of the fluorophores used. In our setup, we could increase the excitation intensity of the donor to increase the FRET intensity, as we are still far from photobleaching. We could also consider using an acceptor with a longer photobleaching half-time to increase the FRET signal.

In this study, we relied on FLIM to obtain the true FRET efficiency of TSMod and used this value to calibrate the intensity-based setup. Because we used CHO-K1 cells on both the Paris-Saclay and Rutgers setups, we assumed that the FRET efficiency expressed in CHO-K1 cells would be the same in both labs. The calibration procedure with a single TSMod construct is rapid, so it can easily be done for each condition or fluorophore. Should a FLIM measurement, or true FRET of a single construct, not be available, the G factor may be obtained using two calibration constructs of unknown FRET efficiency.^24^ However, the use of two separate constructs to determine the G factor is more cumbersome and less precise.^16^ Because the G factor is instrument-dependent, a given setup calibration must be applied to that same setup for FRET measurement. We showed that, in spite of very different G calibration factors on our Paris Saclay and Rutgers setups, due to different filter sets, excitation sources, and intensities, we obtained the same final values for the control VinTL.

Using cheaper cameras allowed us to include two in parallel, avoiding any moving parts in our system. Only two illumination steps are needed, making it faster to acquire the three images required to measure FRET efficiency, at a rate equal to twice the chosen exposure time. This will be an asset for combining FRET efficiency measurement with the application of a time dependent force. An optical tweezer can be added above the FRET excitation path on our setup to simultaneously monitor the force applied on the cell membrane *in vivo* and the FRET efficiency of the VinTS near the point of application of the force.

A limitation of our setup is that it is not currently applicable to thick three-dimensional samples. The FRET calibration and calculation presented here could be applied in a confocal microscope. However, in the case of thin samples, scanning confocal microscopy defeats the purpose of speeding up image acquisition and lowering the cost of the setup. For thick samples, combining our method with light-sheet illumination might be a way to extend it to three-dimensional FRET imaging. Fast 3D FRET imaging could be achieved combining light-sheet microscopy with wide-field FLIM,^12^^,^^14^ although it will require a costly setup.

## Data, Materials, and Code Availability

The raw data supporting the conclusions of this article and the code used for data analysis in this publication are available upon request to the authors, without undue reservation.
