# Supplementary material for: Förster resonance energy transfer efficiency measurements on vinculin tension sensors at focal adhesions using a simple and cost-effective setup
Source: J Biomed Opt. 2023 Jul 11;28(8):082808. doi: 10.1117/1.JBO.28.8.082808 (PMC10335361; doi:10.1117/1.JBO.28.8.082808)
Supplement: Supplementary file 1 [file JBO_028_082808_SD001.pdf]

Supplementary Material

Photobleaching measurements

The half-time of the fluorophores have been measured on the same cells with the same exposure time (0.5 s or 1 s). The exponential decay of the mean intensity value is fitted by $A\ e^{-t/\tau}$ and the half-time is $t_{1/2} = ln(2)\ \tau$.

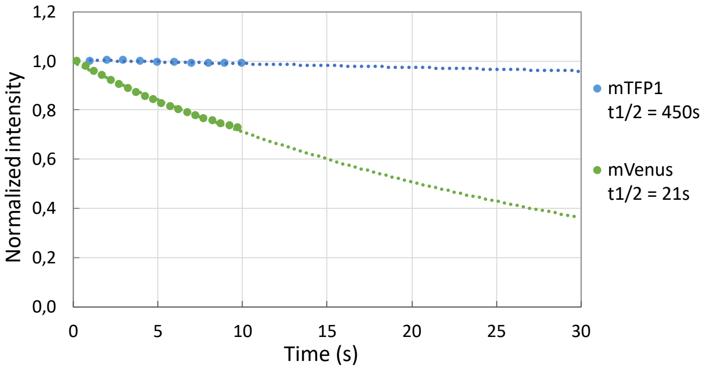

Fig S1: Photobleaching decay half-time measured for CHO-K1 cells transfected with donor or acceptor only. The illumination intensities were $19.0\ mW/mm^2$ for the blue LED and $13.5\ mW/mm^2$ for the green LED.

Intermolecular FRET

Cells expressing very high concentration of tension sensors in their cytoplasm appears to have higher FRET efficiencies likely due to intermolecular FRET phenomenon between distinct FRET-pairs.

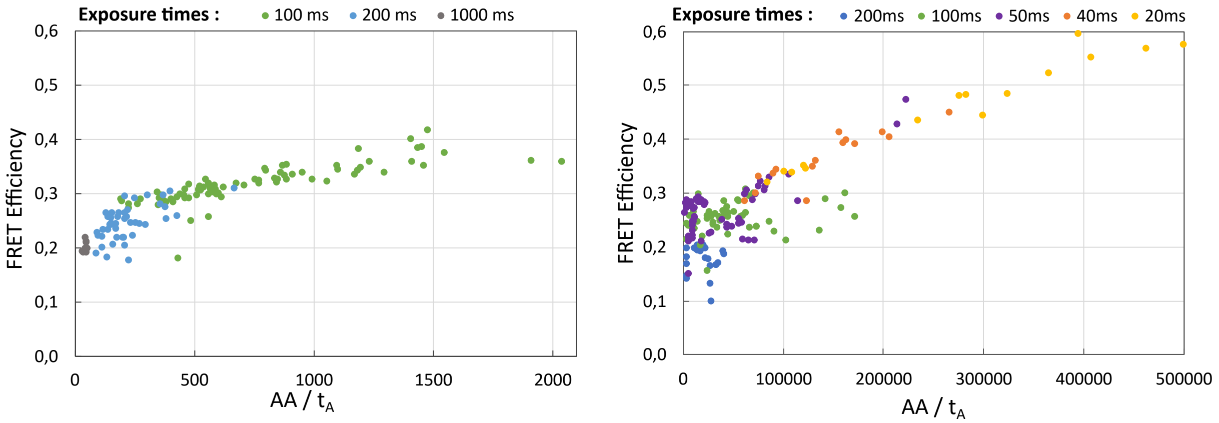

(a) Paris-Saclay (b) Rutgers

Fig S2: Cell-by-cell plotting of the FRET efficiency depending of the level of intensity of the fluorophores given by AA/t_A in grey levels per second in CHO-K1 cells expressing TSMod on the Paris-Saclay (a) and Rutgers (b) setups.

For the calibration, FRET efficiency needs to remain constant, independently of the concentration of fluorophores in the cells. We selected the cells satisfying $AA/t_A < 500$ in grey levels per second at Paris-Saclay and $AA/t_A < 75000$ at Rutgers and used a fixed exposure time of 100 or 200 ms at Paris-Saclay and 50 ms at Rutgers.

Rutgers setup calibration

The calibration protocol and image processing program described in the main text were also used on the Rutgers setup. The bleedthrough constants are $a = 0.573$ and $d = 0.838$. The illumination intensities are $13.3\ mW/mm^2$ for excitation centered at 450 nm and $0.8\ mW/mm^2$ for excitation centered at 514 nm.

The relative concentrations of the donor and the acceptor R can be measured from

$$R = \frac{[D]}{[A]} = \frac{1}{k}\frac{DD + F_c/G}{AA}$$

The main mode value of the 2D histogram plot (Fig S3) corresponds to the cells with a R ratio equals to 1. The data show a second mode value which corresponds to cells expressing an abnormal ratio (Fig S3b). Data are fitted with a sum of two 2D Gaussian models. In order to discard the outliers values, the main operating mode is thus determined using the fit of the main Gaussian. From the coordinates of its center, we obtain : $G = 2.97 \pm 0.10$ and $k = 2.14 \pm 0.08$.

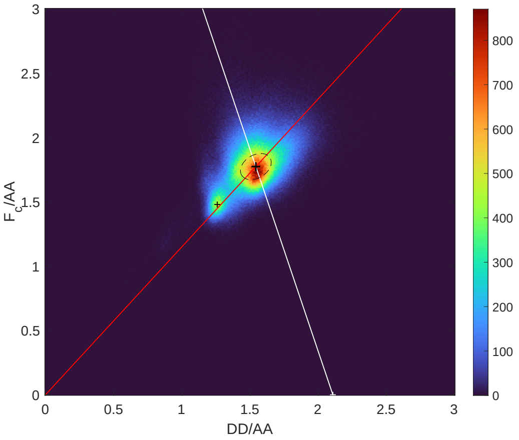

(a)

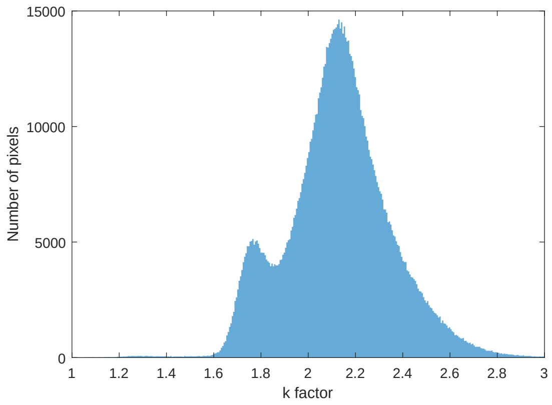

(b)

Fig S3: Calibration of the Rutgers setup. (a) Pixel-by-pixel heatmap of Fc/AA versus DD/AA of CHO-K1 cells expressing TSMod. 61 cells with exposure time of 50 ms, leading to 11 millions pixels. The pixels are fitted with a sum of two 2D Gaussians with an angle : $f(x,y) = A_1\ exp(-(((x-x_1)\ cos(a)+(y-y_1)\ sin(a))/w_{x1})^2 - ((x-x_1)\ sin(a)+(y-y_1)\ cos(a))/w_{y1})^2 + A_2\ exp(-(((x-x_2)\ cos(a)+(y-y_2)\ sin(a))/w_{x2})^2 - ((x-x_2)\ sin(a)+(y-y_2)\ cos(a))/w_{y2})^2$. $R^2 = 0.957$. The black crosses represent the center of the fitted Gaussians and the dashed ellipse is the 95 % confidence bond of the main Gaussian. Its center is given by $x_0 = 1.543 \pm 2.9e-4$ and $y_0 = 1.773 \pm 3.2e-5$. The red and white slopes represent respectively equations (1) and (2) of the main text. (b) Distribution of the k value of each pixel.

Description of the plasmids

Fluorophores			Source
mTFP1	Donor only		B.D.H.
mVenus	Acceptor only		B.D.H.
FRET constructs			
TRAF	TNFα receptor associated factor	mTFP1-TRAF-Venus, low FRET construct (ref 25 of the main manuscript)	B.D.H.
GGS_1		mTFP1-$(GGSGGS)_1$-mVenus, high FRET construct	B.D.H.
GGS_2		mTFP1-$(GGSGGS)_2$-mVenus, high FRET construct (ref 25 of the main manuscript)	B.D.H.
TSMod	Tension sensor module	mTFP1-$(GPGGA)_8$-mVenus, intermediate FRET construct	Addgene #26021
Vinculin tension sensors			
VinTS	Vinculin tension sensor	Sensor module TSMod inserted between the Vh and Vt domain (after amino acid 883) of vinculin	Addgene #26019
VinTL	Vinculin tail-less control	Tail less mutant which cannot bind F-actin or paxillin	Addgene #26020

Table S1: Plasmids. The first five plasmids were kindly provided by Dr. Brent D. Hoffman.

Image processing

An acquisition consists of 3 raw images : DD, DA and AA (Fig. S4). The following steps are done to extract FRET efficiency with a custom Matlab code.

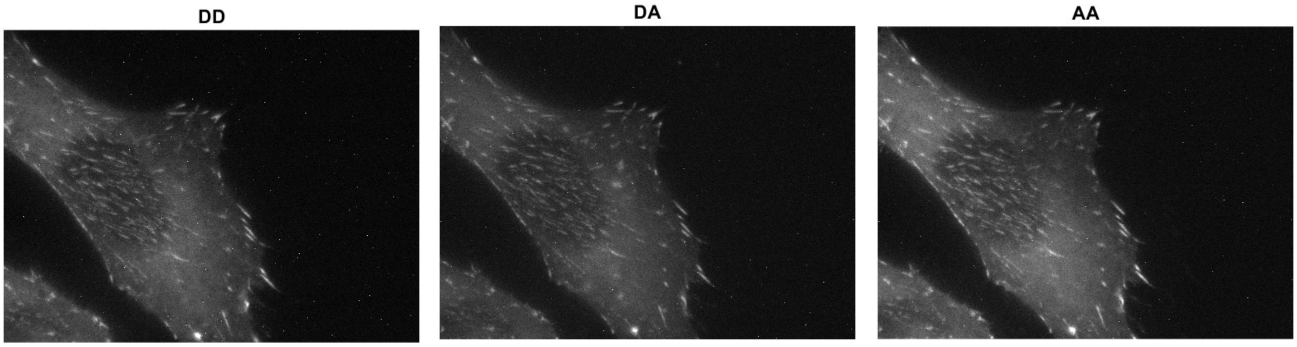

Fig S4: Raw images of DD, DA, DA images of CHO-K1 cells expressing VinTS. $t_D = t_A = 1900ms$.

1. *Background substraction :* A dark region far from the cells or glare effect is manually selected and the mean value of this region is subtracted from each raw image. (in Fig. S4, this region is selected in the upper right corner of the images)

2. *Illumination correction :* Each image is flat-field corrected to account for the illumination profile of each LED that is not perfectly uniform over the field of view. Each image is divided by the normalized illumination profile of the corresponding LED, obtained from 15 to 20 images of a slide with uniform fluorescence (fluorescent marker) that are averaged and smoothed.

3. *Registration :* To adjust the overlap between images from the two separate cameras, a transformation matrix is calculated using the *'imregister'* Matlab function allowing translation, rotation and scale. To compute this transformation, we choose a cell spread over a large part of the field of view, with a good signal-to-noise ratio and FAs without too much background from the cytoplasm. The matrix is then applied to all the cells in the same sample, and a new one is calculated for the next sample.

   This transformation is applied to DD. DA and AA are already perfectly overlaid since they are taken on the same camera without any mechanical movement between the acquisitions. For the image shown in Fig.S5, the transformation matrix is

$$T = \begin{pmatrix} \lambda\cos\theta & \sin\theta & 0 \\ -\sin\theta & \lambda\cos\theta & 0 \\ d_x & d_y & 1 \end{pmatrix} = \begin{pmatrix} 0.98 & 0.001 & 0 \\ -0.001 & 0.98 & 0 \\ 23.5 & 15 & 1 \end{pmatrix}.$$

   λ is the scaling factor, θ the rotation angle, d_x and d_y the translations in pixels.

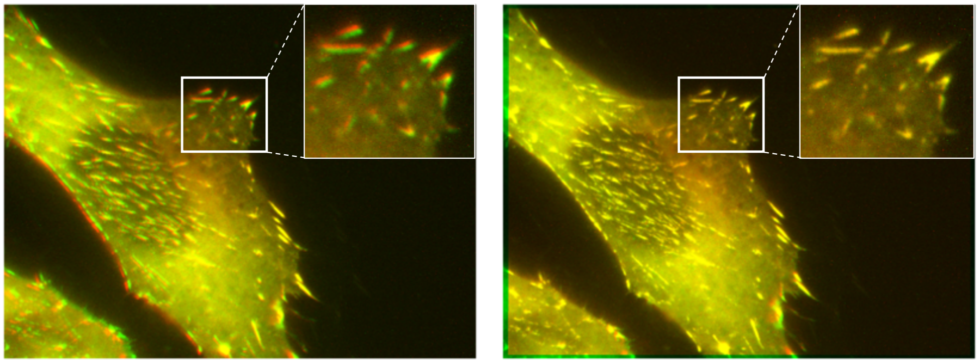

(a) Raw images (b) After registration

Fig S5: Superposition of DD in red an AA in green. The zoom on the white square area shows the improved overlap after registration.

4. *Region of interest :* The ROI is manually selected to isolate each cell (in the following steps, the main cell was selected).

5. *Mask definition :*

- For mTFP1, mVenus and TSMod: Cells are isolated from the background pixels by a simple thresholding.
- For VinTS and VinTL, we calculate a mask to isolate focal adhesions: an algorithm based on a rolling ball background subtraction is applied on each channel.

  The steps of this algorithm are depicted in Fig.S6 on an AA image with a profile on the left to illustrate the effect. The background calculated from the rolling ball averaging (ref 16 of the main manuscript) is subtracted from the image. The 'ball' diameter is chosen bigger than the larger FAs, at 20 pixels. Once this background is subtracted from the images, a Gaussian filter with $\sigma = 3$ pixels is applied to smooth the images and remove the isolated pixels due to noise. Then a binary mask is created by thresholding. This requires cropping of the image edges by half the ball diameter.

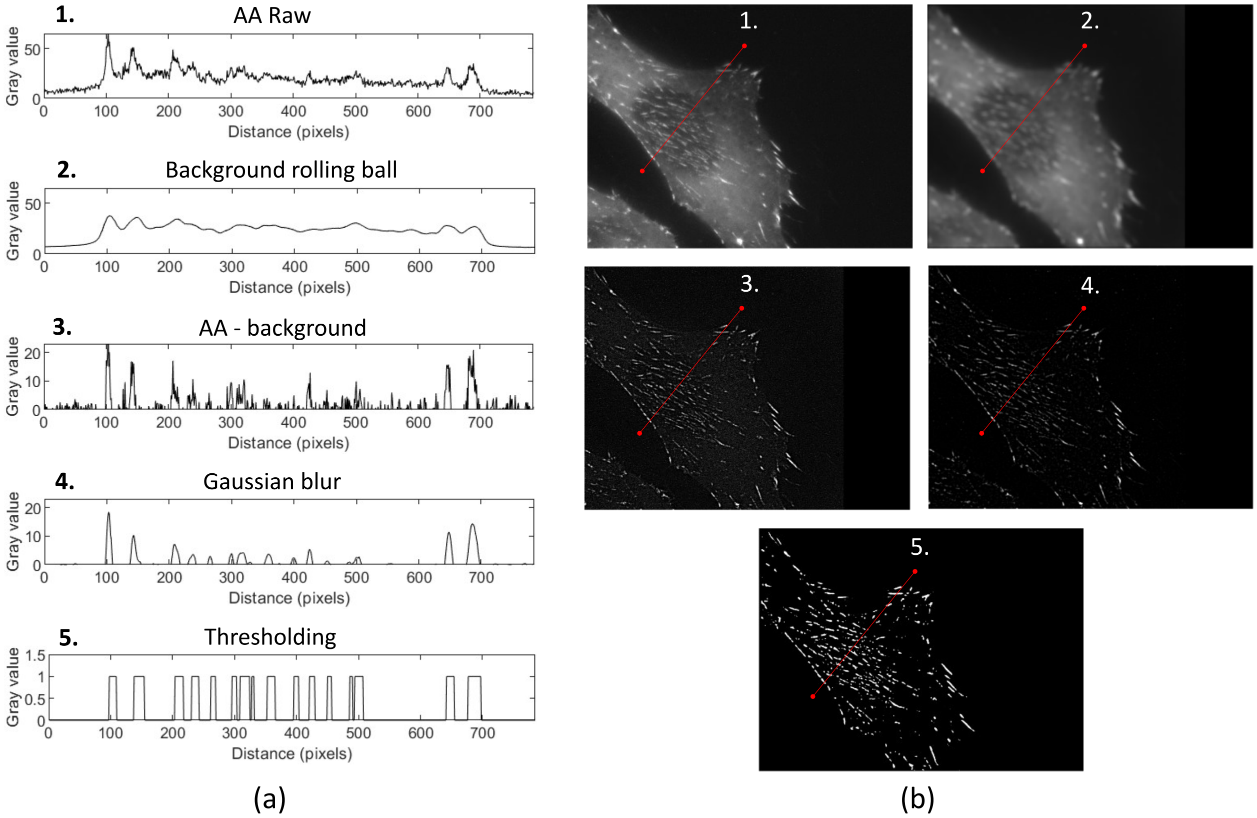

Fig S6: Image processing steps illustrated on the AA image: 1. Raw image 2. Background given by the rolling ball algorithm 3. AA after rolling ball background subtraction 4. Gaussian blur 5. Binary mask. (a) are profiles along the red line of the images (b).

This processing is done independently on the DD, DA and DA images and the final mask keeps only the coinciding pixels from the 3 masks (Fig. S7). This helps avoid

edge effects during the mathematical operations needed to extract FRET efficiencies, due to imperfect registration or to slight motion of the cell between acquisitions.

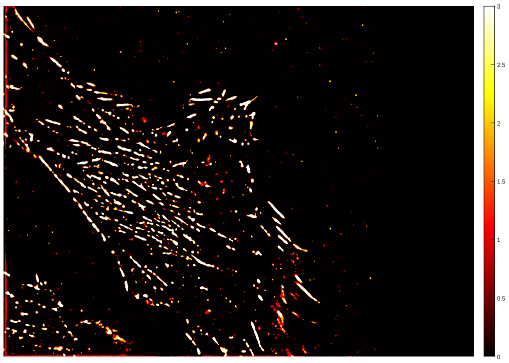

Fig S7: Overlay of the 3 masks for each channel. The final mask is the coincidence of the 3 masks, appearing as white pixels.

6. *Masking the images :* The final FAs mask and the ROI are applied to DD, DA and AA images, after steps 1 to 4 of the image processing. Note that the background subtraction remains that of step 1, and not the rolling ball background used to define the mask.

7. *Corrected FRET :* The corrected FRET is calculated by : $F_c = DA - a\ AA - d\ DD$ with $a = 0.054$ and $d = 0.384$ (Fig. S8a). The measurement of a and d factors is based on images which have been through steps 1-4 of image processing.

8. *FRET efficiency :* FRET efficiency is given by : $E = \frac{F_c/DD}{G+F_c/DD}$ with $G = 1.54$ (Fig. S8b).

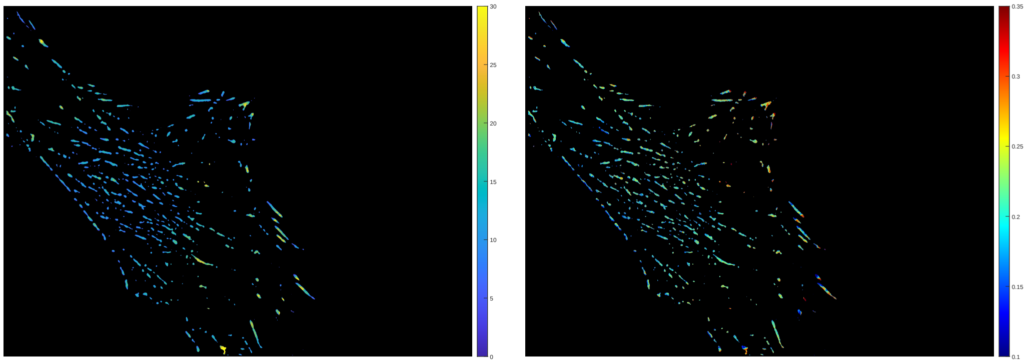

(a) Corrected FRET F_c (b) FRET efficiency E

Fig S8: Pixel-by-pixel plot of the corrected FRET and of the FRET efficiency.

9. *Segmentation :* Each focal adhesion is segmented from the binary mask (step 5) using the *'bwconncomp'* Matlab function with a connectivity $cc = 8$. The segments that contain less than 15 pixels are discarded. We average the efficiency over each segmented FA. Other parameters of the FAs could be extracted from the segmentation : size, intensity, corrected FRET, FRET efficiency, shape, ...

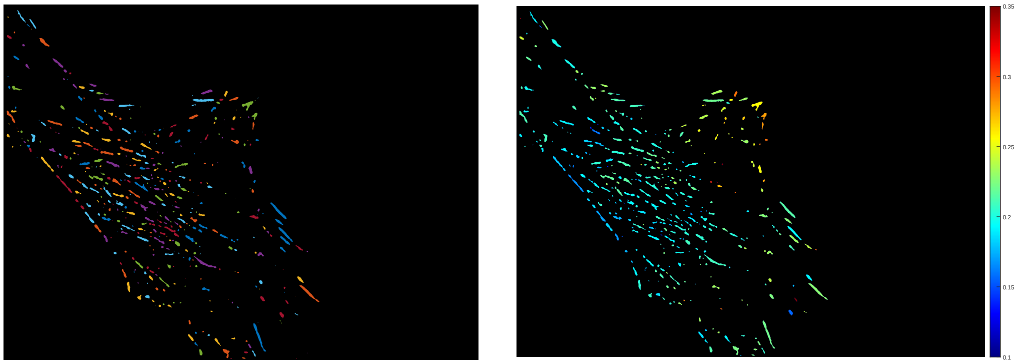

(a) Segmentation of the mask (b) FRET efficiency

Fig S9: (a) Result of the segmentation. Colors are used to identify each segmented area, but do not represent FRET. (b) FRET efficiency averaged over each segmented FA.

Low fluorescence thresholding

When analyzing the VinTL and VinTS data, we discarded focal adhesion sites that had fluorescence signal below a certain threshold. We defined this threshold on the AA image, since this image does not depend on FRET efficiency. As shown in Fig. S10a, the average FRET efficiency measured for VinTL varies by less than 2% due to the choice of threshold, a systematic effect that is smaller than the standard deviation. We chose a threshold of 5 grey levels for our exposure time of 1.9 seconds, which corresponds to a situation where the DA signal is equal to its background. DA is the noisiest signal, with more autofluorescence background than the DD and AA images. The standard deviation does not increase much if we choose a lower threshold, showing that we could apply our measurement to noisier data. The same conclusions can be drawn on the VinTS data, as shown in Fig. S10b. The same criterion (signal equal to background for DA) was applied to the Rutgers data, corresponding to a threshold in AA of 300 grey levels for 1 second exposure time.

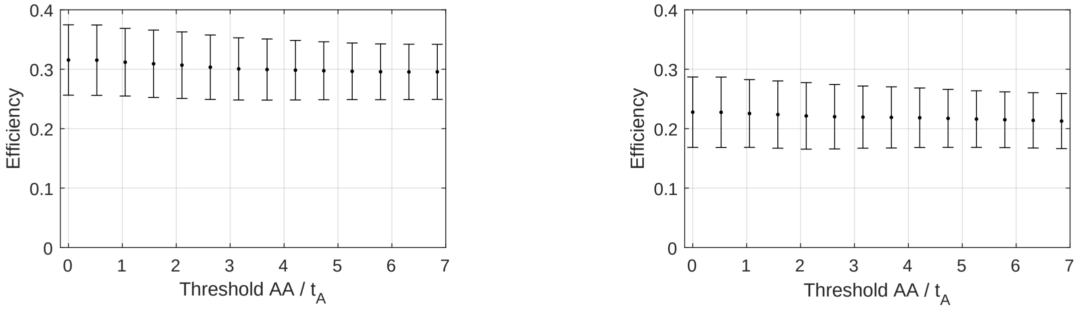

(a) VinTL (b) VinTS

Fig S10: Average FRET efficiency of VinTL and VinTS as a function of the threshold on the AA signal. Error bars are standard deviations on all the selected focal adhesions above threshold.
